# Impact of Maternal Education about Complementary Feeding on Their Infants' Nutritional Outcomes in Low- and Middle-income Households: A Community-based Randomized Interventional Study in Karachi, Pakistan

**Published:** 2014-12

**Authors:** Ali Faisal Saleem, Sadia Mahmud, Naila Baig-Ansari, Anita K.M. Zaidi

**Affiliations:** ^1^Department of Paediatrics and Child Health, Aga Khan University, Karachi, Pakistan; ^2^Department of Community Health Sciences, Aga Khan University, Karachi, Pakistan

**Keywords:** Complementary feeding, Infants' growth, Infants' nutrition, z-scores, Pakistan

## Abstract

This cluster-randomized interventional trial at peri-urban settings of Karachi was conducted to evaluate the impact of maternal educational messages regarding appropriate complementary feeding (CF) on the nutritional status of their infants after 30 weeks of educational interventions delivered by trained community health workers. Mothers in the intervention group received three education modules about breastfeeding (BF) and appropriate CF at a baseline visit and two subsequent visits 10 weeks apart. The control group received advice about BF according to national guidelines. Infants' growth [weight, length, and mid-upper arm-circumference (MUAC), stunting, wasting, and underweight] were measured at four time points. At the end of the study, infants in the intervention group had a higher mean weight of 350 g (p=0.001); length of 0.66 cm (p=0.001), and MUAC of 0.46 cm (p=0.002) compared to the controls; proportionate reduction of stunting and underweight were 10% (84% vs 74%; OR_adj_ 8.36 (5.6-12.42) and 5% (25% vs 20%; OR_adj_ 0.75 (0.4-1.79) in the intervention compared to the control group. For relatively food-secure populations, educational interventions about appropriate CF to mothers had a direct positive impact on linear growth of their infants.

## INTRODUCTION

Malnutrition is a major preventable risk factor for disability and premature mortality of children in developing countries. By eliminating malnutrition, it is estimated that child mortality could be decreased by more than half and the burden of disease by more than 20% in developing countries (1). According to the WHO, inappropriate feeding practices are one of the major causes of high morbidity in young infants of the developing world (2). There is increasing evidence for the positive impact of feeding-counselling on energy and nutrient intake and on the growth of children aged less than two years (3,4).

Complementary feeding plays an important role in infant growth, intellect, and development while inappropriate complementary feeding practices are associated with malnutrition. Maternal education in complementary feeding has some role in Infants' linear growth and WHO growth parameters in children of developing countries.

Introduction of complementary food (CF) at six months of age helps in filling the gap between nutritional needs and the amounts provided by breastmilk. The timing of the introduction of CF is important because too early or delayed initiation may be associated with adverse consequences. Infants develop more respiratory illnesses and eczema if CF is started earlier than six months (5). This also exposes infants to pathogenic microbes contained in unhygienic food or water. Conversely, delay in introducing CF beyond six months may lead to an inadequate nutrient intake (6,7), delay in development of eating behaviour, and oral-motor skills (chewing, swallowing, and speaking) (8). Delayed introduction of semi-solid food after the age of 10 months is associated with an increased risk of feeding difficulties (9).

Promoting optimal complementary feeding practices in developing countries is a global health priority (10). Pakistan still suffers a high infant mortality rate (78/1,000), and progress in meeting child survival goals has been slow (1,11). According to the UNICEF's Pakistan National Nutritional Survey 2011, the prevalence of overall stunting in Pakistan in the under-5 population is 43.7%, wasting 15%, and underweight 31.5% (12). Multiple variables, i.e. peculiar food practices in diet by various cultural and ethnic variabilities, delayed or early initiation of CF, maternal knowledge and understanding regarding CF and Infants' diet, family planning practices, and birth spacing, have been identified as risk factors of stunting and under-five malnutrition. CF practices in Pakistan are not optimal, and these may be inadequate in energy, proteins, and micronutrient quality and/or quantity. Furthermore, CFs are often prepared, stored or fed in ways that increase the risk of contributing to morbidity and mortality of children. We have observed that children living in low- and middle-income (fourth wealth quintile) urban areas that are not food-insecure also have high stunting rates (author's unpublished data). An estimated 23% of the population of Pakistan has been classified in this quintile (13) and could benefit from appropriate feeding advice through a variety of communication strategies. Maternal education regarding CF may improve nutritional status of infants in households that are relatively food-secure but lack knowledge about appropriate CF practices (14). With rapid urbanization in South Asia, increase in food-commodity prices and declining fertility, a significant proportion of urban and rural households now have access to sufficient calories but inadequate information on appropriate food choices. This study evaluates the impact of maternal educational strategies regarding appropriate CF for infants in low- and middle-income urban households in Karachi and assesses their impact on nutritional status after 30 weeks of educational intervention.

## MATERIALS AND METHODS

Karachi is a metropolitan city divided into 18 towns. We conducted this cluster-randomized interventional trial at Bhains Colony (Cattle Colony), a peri-urban setting of Karachi located in Bin Qasim Town, with a total population of 56,178 according to the 2007 census. Most people in this locality are from low- and middle-income households with dairy farming as the major local industry. The study population comprises mothers of infants aged 10-20 weeks, who were either exclusively or partially breastfed but had not started CF or had recently started (less than one week prior to enrollment), and lived in the study area. Infants were excluded if they were already below the 5th percentile in WHO growth charts on weight-for-age at baseline, had a history of two or more hospital admissions at the time of enrollment (each hospital stay >7 days), had serious congenital anomalies (cleft palate, congenital heart disease, neural tube defect), other chronic conditions impairing feeding (e.g. cerebral palsy) or the presence of acute illness, and/or severe anaemia, which required urgent hospitalization at the time of enrollment. Socioeconomic status was ascertained by using household asset score (by assessing the assets and utilities, including electricity, natural gas connection, telephone/mobile phone, automobile, cycle, television, refrigerator, separate water supply, and separate kitchen) as also used previously (15,16). Each of these was given scores (1=yes, 0=no), and a minimum 6/9 was required to be eligible for enrollment. None of our study participants was excluded on the basis of underscoring of household commodities.

### Sampling technique and recruitment

In 2007, the Department of Paediatrics and Child Health of Aga Khan University conducted a door-to-door census of the locality as the baseline information was needed for studies on aetiology of diarrhoea. We updated the existing census and expanded the line-listing by enrolling some more areas of the town. Ten geographically-distinct areas were randomly assigned by using random number table to educational intervention and control. Potential participants were enrolled in the study by using eligibility criteria and a screening questionnaire as mentioned above. Household demographic information was obtained at enrollment. Weight (kg), length (cm), MUAC (cm), and z-scores [underweight or weight-for-age (WAZ), stunting or length-for-age (LAZ), and wasting or weight-for-length (WLZ)] were the dependent variables while socioeconomic status, family characteristics and practices (boiling water, maternal and paternal age, educational status, and native language), and Infants' characteristics (gender, age at baseline, vaccination status, and initial feeding practices) were the independent variables.

### Intervention at home visits

Educational interventions were offered at participants’ homes. There were a total of four visits (baseline and three subsequent visits at 10 weeks interval). The intervention group had specific CF education sessions given to mothers in the first three visits. Ten important key messages were developed based on recommended practices (WHO/UNICEF 2000 and 2006) (3,17). These messages were adapted for the local population according to locally-available CF choices. The interventions were packaged into sessions as follows: (i) baseline visit covered the importance of breastfeeding, its continuation for the first two years of life and the importance of CF initiation at 6 months of age. The session also included the importance of handwashing and general hygiene; (ii) second teaching session included breastfeeding promotion, consistency in complementary food, selection of initial complementary food, and education in age-related complementary food; and (iii) the third teaching session covered all previous teaching sessions, along with advice on promoting protein-based and iron-rich foods. Breastfeeding promotion and education on the preparation of oral rehydration solution were imparted to both groups. Verbal, pictorial and demonstration techniques were used in each teaching session with an average duration of 15-20 minutes. In order to minimize the bias, educational session was conducted, and Infants' anthropometric measurements were taken by different teams and on different days.

### Training of research team members

Two female research assistants (with at least 14 years of schooling) and two female community health workers (with at least 10 years of schooling) were involved in this study. The research team received two weeks of training. Pre- and post-test were administered to the trainees to assess the change in their knowledge and their understanding regarding data-collection procedures. Trainees were specifically trained in CF education. The principal investigator conducted the entire training and developed the training materials.

### Sample-size calculation

Sample-size was calculated using repeated measures design with four time points. A sample of 59 subjects in each group was needed to detect a difference in weight of 250 g and a difference in length of 1 cm between infants in the intervention and control group, with 5% significance level and 80% power. Thirty subjects in each group were needed to detect a difference of 0.5 cm in MUAC between infants in the intervention and the control group, with 5% significance level and 80% power. We took a design effect of 1.25 and adjusted for cluster effect by multiplying the maximum sample of 59 with 1.25. Hence, our final sample-size was 74 in each group.

### Statistical analysis

The data were analyzed using Statistical Package for Social Sciences (SPSS) (version 19), SAS 9.1, and WHO Anthro 2005 software. Baseline characteristics were compared between the groups, using chi-square test for categorical variables and *t*-test for continuous variables. Infants with readings from at least first two visits were considered for statistical analysis.

Comparisons of variables, including weight, length, and MUAC between groups across time at baseline, 10, 20, and 30 weeks were made by repeated-measures analysis of variance (ANOVA), using PROC MIXED on SAS (SAS Institute, 2004, Release 9.1). A p value <0.05 was considered statistically significant. PROC MIXED analyzes data in a relational (longitudinal) format, gives flexibility in modelling the variance-covariance structure (3), and allows for a variable number of follow-ups for different subjects. The selection of the variance-covariance structure was made from the following commonly-used patterns (9): (i) auto-regressive order 1 (AR-1) and (ii) compound symmetry (CS). AR-1 was used in order to model the covariance structure. The analyses were adjusted for gender, age (in days) at baseline, vaccination status at baseline as well as father's and mother's educational status. Interactions of variable ‘group’ with all variables in the model were also assessed for significance.

The z-scores (underweight, stunting, and wasting) were calculated by using WHO Anthro 2005 software. A cutoff of −2SD (standard deviation) was used in order to categorize children as underweight, stunted, or wasted. We conducted a generalized estimation equation (GEE) analysis to assess the differences in proportion of underweight, stunting, and wasting between the intervention and the control group. Analyses were adjusted for age in days at baseline, vaccination status at baseline as well as father's and mother's educational status.

### Ethical approval

The study was initiated after approval from Ethical Review Committee of Aga Khan University Hospital, Karachi (102–PED/ERC–08). Formal permissions (written and verbal) were also taken from the Nazim (town administrator) of the study area. The trial was registered at www.clinicaltrial.gov (NCT01128517).

## RESULTS

### Comparison of sociodemographic features of study population

**Figure 1.**
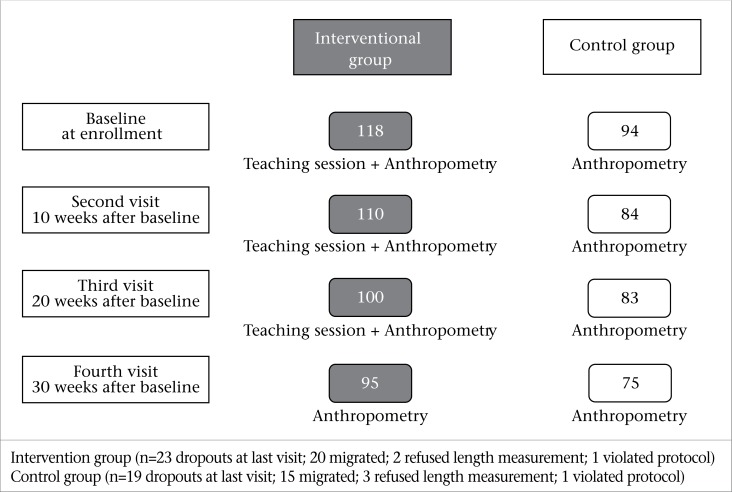
Flow diagram of the study

A total of 212 infants (118 in the intervention and 94 in the control clusters) were recruited in the study. One hundred ninety-four infants (intervention 110 and control 84) were considered in the final statistical analysis **(**Figure 1). Overall, there were 95 remaining infants in the intervention, and 75 in the control cluster at the end of the study (fourth visit). Baseline characteristics are reported in Table 1. More than two-thirds (71%) of the infants were exclusively breastfed at the time of enrollment. Infants in control clusters were slightly older at baseline (p=0.03) and more likely to be vaccinated (p=0.03). There was no difference in the mean weight (p=0.27) and mean length (p=0.62) at baseline between the two groups; however, infants in the control clusters were less wasted (7% vs 13%) and underweight (16% vs 26%) compared to intervention clusters. Mean MUAC of infants in the intervention clusters was greater than that in the control (12.5±0.9 cm vs 12.2±1.1 cm; p=0.03). Literacy rate among parents in both intervention and control clusters was low. Overall, 53 (47%) mothers and 36 (32%) fathers were non-literate in the intervention clusters and 56 (67%) mothers and 49 (58%) fathers in the control clusters.

### Linear growth difference among intervention clusters and controls

**Figure 2.**
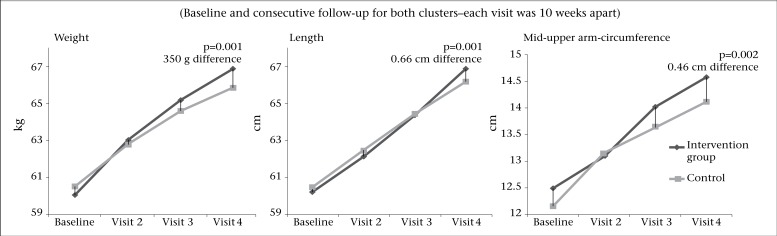
Mean weight, length, and MUAC of infants in the intervention and control clusters

Figure 2 shows the visits and mean measurements of weight, length, and MUAC during and at the end of the study. At the end of the study, the mean weight of infants in the intervention clusters was 350 g greater than that of infants in the control cluster (p=0.001). Infants in the intervention clusters were 0.66 cm (p=0.001) taller, and their MUAC increased by 0.46 cm compared to controls (p=0.002) at final visit (Table 2).

### Differences in WHO growth parameter among intervention clusters and controls

**Figure 3.**
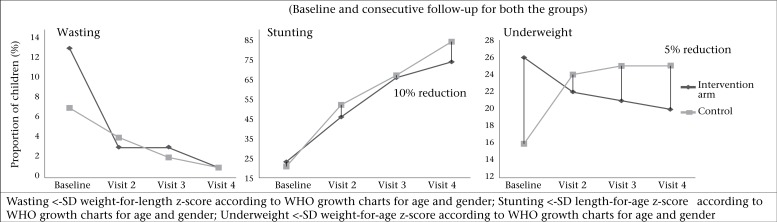
Proportion of wasting, stunting, and underweight in infants in the intervention clusters receiving nutritional education compared to infants in the control clusters

Proportion of wasting, stunting, and underweight at baseline and on subsequent visits were plotted (Figure 3). There was a 6% reduction (baseline 26% vs endline 20%) in the proportion of underweight infants in the intervention group and a 9% increase (baseline 16% vs endline 25%) in underweight infants in the control clusters at the end of the study. Thirteen percent infants in the intervention and 7% infants in the control group were wasted at the beginning of the study. There was a 12% reduction in wasting in the intervention group by the end of the study but there was no significant difference between the groups at the end of the study (OR_adj_=1.42; 95% CI 0.56-3.58). At baseline, almost equal proportions of infants were stunted in both the groups. The infants in the control group were eight times more stunted compared to intervention group at the end of the fourth visit (OR_adj_=8.36; 95% CI 5.60-12.42). However, the GEE regression analysis indicated that there was no significant difference in stunting between the two groups at any visit (OR_adj_=0.74; 95% CI 0.44-1.25) (Table 3).

**Table 1.** Baseline characteristics of infants in nutritional education in the intervention and control clusters

Characteristics	Intervention (n=110)	Control (n=84)	p value
Infants' characteristics			
Age in days (mean±SD)	106.5±22.4	113.7±24.5	0.03
Mode of delivery (SVD), n (%)	99 (89)	70 (83)	0.17
Vaccinated, n (%)	67 (60)	49 (58)	0.03
Gender			
Male, n (%)	65 (59)	54 (64)	0.25
Birth place			
Hospital, n (%)	73 (66)	48 (57)	0.14
Initial feeding practices, n (%)			
Exclusive BF BF+Buffalo/Cow's milk BF+formula Formula	83 (75) 9 (8) 11 (10) 7 (6)	54 (64) 11 (13) 13 (16) 4 (5)	0.12
Infant measurements			
Weight (kg) (mean±SD) Length (cm) (mean±SD) MUAC (cm) (mean±SD)	5.7±0.8 60.2±3.4 12.5±0.9	5.8±0.7 60.4±3.3 12.2±1.1	0.27 0.62 **0.02**
% of stunting at first visit % of underweight at first visit % of wasting at first visit	21 26 13	20 16 7	0.85 0.08 0.21
Family and other factors			
Head of family (parent/s), n (%)	34 (36)	24 (32)	0.36
Father's age (mean±SD)	32.3±7.1	31.1±5.5	0.22
Mother's age (mean±SD)	26.9±4.3	26.7±4.2	0.78
Father's educational status, n (%)			
Non-literate	36 (32)	49 (58)	0.001
Mother's educational status, n (%)			
Non-literate	53 (47)	56 (67)	0.01
Number of family members (mean±SD)	9.0±4.2	10.0±3.8	0.32
Home ownership, n (%)	73 (66)	78 (79)	0.06
Earning members in the family, n (%)			
1 2 >2	36 (32) 42 (38) 33 (30)	33 (39) 30 (36) 21 (25)	0.58
Boil water for drinking, n (%)	12 (13)	6 (7)	0.10
Cemented roof, n (%)	105 (95)	78 (93)	0.50

SD=Standard deviation; SVD=Spontaneous vaginal delivery

## DISCUSSION

Inadequate nutrition during the initial two years of life is an important preventable risk factor of childhood morbidity and mortality (18). Unavailability of food is not the sole cause of malnutrition. Feeding practices in a community (19), lack of awareness and knowledge about age-appropriate food items, food quantity and frequency also contribute significantly to poor nutritional status of a child. In our study, we selected low- and middle-income rather than food-insecure households in order to concentrate on the care aspect of malnutrition. The socioeconomic status of households was assessed by criteria used in other studies (15,16). Although there are complex models to assess socioeconomic status, we modified and used a simpler screening tool to identify low- to middle-income households for the recruitment of study subjects. We also excluded high-risk infants, i.e. severely malnourished and severely anaemic as these needed vigorous interventions. However, no infant included in our study was found to be severely anaemic.

We chose to conduct the teaching sessions at homes as the most convenient location for the mother as was done elsewhere (16,20-22). and kept the number of visits to a minimum (only 4) with a view to scaling up using the Pakistan Government's Lady Health Worker (LHW) Programme. The LHW Programme employs approximately 100,000 women who make household visits, conducting primary healthcare and family counselling. Various facilities (community health clinics, immunization clinics, hospitals, and visiting homes) were used for the educational intervention (23). We held our educational sessions at the participants’ homes. This approach gave us more opportunity and time to interact with and educate mothers and, ultimately, showed a positive impact on the Infants' linear growth. We educated mothers to use animal-based food, i.e. eggs, butter, liver, and chicken/beef. Studies that used locally-appropriate complementary food items rich in animal proteins and micronutrients showed a positive impact in linear growth of infants (22,24,25).

Imdad *et al.*'s (26) pooled analysis and systemic review of the impact of maternal education on complementary feeding in developing countries showed a mean weight gain of 300±260 g and a mean length gain of 0.49±0.5 cm. There are studies which showed no to very minimal mean weight difference between the intervention and control groups at the end of educational intervention (20,22,24). However, the maximum impact of maternal education on infant's mean weight gain of 940 and 570 g respectively were shown by Guldan *et al*. (25) and Roy *et al.* (27). Infants' length gain is much slower than the weight and requires a longer-duration follow-up to observe a difference after an intervention. Studies which are of short-duration follow-up failed to show any significant difference between educational intervention group compared to controls (mean length gain −0.2 to 0.15 cm) (20,22,24,27). Penny *et al*. (18) reported a 0.95 cm length gain after 18 months of educational intervention while Bhandari *et al.*'s (20) intervention groups were 0.3 cm taller than controls after 1 year of educational intervention. Duration of our educational intervention was 30 weeks, and at the end of the study, infants in the intervention cluster were 350 g heavier and 0.66 cm taller compared to control infants. Significant difference in linear growth parameters in the intervention group could be due to the fact that we used age- and locality-appropriate complementary food-related one-on-one teaching technique, educating the subjects at their own homes, in a study population not financially constrained and with well-spaced teaching sessions.

**Table 2.** Linear growth measurements at baseline and at consecutive follow-up, along with Mixed Model Analysis for weight, length and MUAC measurements

	Intervention clusters	Control clusters	Mean difference	p value†
Weight (kg)				
Baseline	5.74±0.86	5.90±0.78	350 g	0.001
Visit 2ˆ	6.78±0.87	6.71±0.83		
Visit 3ˆ	7.53±0.85	7.33±0.89		
Visit 4ˆ	8.13±1.13	7.78±0.86		
Length (cm)				
Baseline	60.23±3.43	60.50±3.32	0.66 cm	0.001
Visit 2ˆ	62.15±3.46	62.46±3.32		
Visit 3ˆ	64.38±3.29	64.48±3.19		
Visit 4ˆ	66.86±3.41	66.20±3.09		
MUAC (cm)				
Baseline	12.50±0.97	12.20±1.06	0.46 cm	0.002
Visit 2ˆ	13.30±1.01	13.14±1.14		
Visit 3ˆ	14.02±1.02	13.65±1.08		
Visit 4ˆ	14.59±1.05	14.13±0.99		

ˆBaseline is the reference;

†Final model (group, visit, age in days, and group visit) was adjusted for gender, vaccination status, father's and mother's education

Stunting starts increasing after the age of 3 months and peaks at 3 years (28). According to the National Nutritional Survey, 43% of Pakistani children younger than 5 years were moderately to severely stunted. We observed a high rate of stunting in our intervention and control clusters at the end of the study (74% vs 84% respectively). The reported reduction in stunting after educational intervention ranges between 5% and 11% (18,20,28). Our intervention cluster was 10% less stunted compared to the controls at the end of the study. High stunting rates despite intervention indicate that CF education alone may not be enough and, other factors, such as lack of exclusive breastfeeding, frequent diarrhoeal illnesses, etc. also take a toll on infant growth. Interestingly, studies which used food and/or micronutrient supplementation, along with educational intervention, also failed to show any impact on reduction of stunting (23).

Childhood underweight is recognized as an important public-health problem because of its crucial effects on human performance, health, and survival (29). It has been estimated that about 53% of all deaths in young children are attributable to underweight (30). Stunting and underweight are frequently associated with repeated exposure to financial insecurity, poor sanitation, and the interactive effects of poor energy and nutrient intakes plus infection (31,32). Educational intervention on complementary feeding has some effects on underweight. There is little impact of educational intervention in reducing underweight (2-6% reduction in underweight) (18,25,28). There was a gradual reduction in underweight pattern in our intervention group compared to the control group (Figure 3). Inappropriate feeding practices, along with other factors (vitamin A, D deficiency, measles, and worm infestation) are associated with wasting (33). At baseline, about 13% of infants in the intervention group and only 7% infants in the control group were wasted. However, at the end of study, only 1% of children were wasted in both clusters.

### Strengths

The findings of our study support CF education to the mothers because of its positive impact on linear growth of infants. CF education is relevant for the low- to middle-income households where access to food is not a constraint, and it is the knowledge about CF that is deficient. Randomization was performed on geographical areas to decrease confounding effect among the groups. Our response rates were high. One reason could be that CF education was given at the homes of study participants. We used a mixed model approach for analysis that deals with the missing values in the data. All the models were adjusted for age in days, vaccination status, and parents’ educational status. Measurements were done, and teaching education was provided by different teams in an attempt to minimize measurement bias. Our educational sessions were face-to-face and interactive, which eventually produced a positive impact on linear growth of infants in the intervention group. We used interventional messages based on standardized documents of WHO/UNICEF 2000 and 2006 manual and adapted those according to local needs (17). We measured all the possible linear growth parameters (weight, length, and MUAC) and computed z-scores (wasting, stunting, and underweight). Previous studies on educational intervention did not calculate all the linear growth parameters or impact on z-scores.

### Limitations

Our study has some limitations. Parents’ educational status, vaccination status, MUAC, and mean age of infants at baseline were different between the two groups; however, the mixed model and GEE analyses allowed us to adjust for these variables in the final models. The study focused on households that did not seem to have financial constraints on accessing food but this strategy does not address the high malnutrition rates prevalent in the food-insecure, highly-impoverished households. Our follow-ups were well-spaced but extending the time periods or introducing another follow-up may ensure even better compliance by study populations and may have more beneficial effect on linear growth in infants with mothers receiving special educational intervention on complementary foods.

**Table 3.** Proportion of stunting, wasting, and underweight at baseline and subsequent visits and their generalized estimation equations, logistic regression analysis

Variable	Interventional clusters n (%)	Control clusters n (%)	Adjusted odds ratio*	95% Confidence interval
Underweight†				
Baseline	29 (26)	14 (16)	2.18	1.02- 4.61
Visit 2ˆ	25 (22)	22 (24)	0.94	0.43-1.92
Visit 3ˆ	22 (21)	24 (25)	0.85	0.33-2.20
Visit 4ˆ	19 (20)	24 (25)	0.75	0.40-1.79
Stunting				
Baseline	25 (23)	18 (21)	0.74	0.44-1.25
Visit 2ˆ	55 (46)	44 (52)	3.67	2.64-5.09
Visit 3ˆ	69 (66)	58 (67)	8.37	5.58-12.42
Visit 4ˆ	73 (74)	65 (84)	8.36	5.60-12.42
Wasting				
Baseline	14 (13)	6 (7)	1.42	0.56-3.58
Visit 2ˆ	3 (2)	3 (4)	0.43	0.14-1.26
Visit 3ˆ	4 (3)	2 (2)	0.23	0.09-0.61
Visit 4ˆ	1 (1)	2 (1)	0.10	0.02-0.46

*Adjusted for age in days at baseline, vaccination status at baseline, father's and mother's education;

ˆChange in parameters in the intervention versus the control group compared to baseline;

†Significant interaction between groups and visit

### Conclusions

Maternal education has a direct positive impact on linear growth of infants in food-secure population. Mothers who received educational intervention had heavier, taller, and healthier infants at the end of the intervention compared to mothers who did not receive education on complementary foods in population without any visible financial constraints to the purchase of foods.

### Recommendations and policy implications

CF education for mothers has an important impact on Infants' growth in food-secure population. Hence, we recommend that this be incorporated into the Lady Health Workers Programme of Pakistan and more effective mother and child health be made possible. For food-insecure populations, food supplementation, along with CF education, may have an impact.

## ACKNOWLEDGEMENTS

We thank Dr. Salim Allana, Ms Rizwana, Ms Mahjabeen, and all other study staff for their assistance, cooperation and utmost support throughout this project. This study was funded by Aga Khan University Research Council and NIH-Fogarty research training fund. Dr. Ali Faisal Saleem received research training support from the Fogarty International Center (1 D43 TW007585-01) of the National Institutes of Health, USA.
